# 如何认识和处理肿瘤异质性

**DOI:** 10.3779/j.issn.1009-3419.2018.09.11

**Published:** 2018-09-20

**Authors:** 睿 钟, 慧 李, 爽 张

**Affiliations:** 1 130012 长春，吉林省肿瘤医院肿瘤转化医学实验室 Medical Oncology Translational Research Lab, Jilin Provincial Cancer Hospital, Changchun 130012, China; 2 130012 长春，吉林省肿瘤医院胸部肿瘤内科 Department of Toracic Oncology, Jilin Provincial Cancer Hospital, Changchun 130012, China

**Keywords:** 肿瘤异质性, 精准医疗, 驱动基因, 新技术, Heterogeneity, Precise medicine, Driver genes, Novel technology

## Abstract

肿瘤异质性是恶性肿瘤的特征之一，可使肿瘤的生长速度、侵袭与转移、药物敏感性、预后等各方面产生差异。肿瘤驱动基因和靶向药物的发现发展开启了战胜肿瘤的希望之门，然而异质性的存在又让肿瘤治疗陷入了难以攻克的困境。在肿瘤复发、进展演化的过程中肿瘤异质性如影随形，纷繁复杂。凭借不断进步的检测技术认识和理解肿瘤异质性，针对肿瘤异质性的原因和表型，定制治疗方案已成为当今精准医疗领域的重点范畴。本综述对肿瘤异质性进行了分析和探讨，从而更好的帮助我们了解肿瘤异质性，有利于我们通过多种手段对抗肿瘤异质性。

## Abstract (en)

Tumor heterogeneity is one of the characteristics of malignant tumors, which can cause differences in tumor growth rate, invasion, migration, drug sensitivity and prognosis. Discoveries and development upon on tumor-driver genes and targeted therapy paved the way on dealing with cancer diagnosis and treatment. However, the existence of tumor heterogeneity makes malignant tumor more hardly to overcome. It is generally present and far more complicated during the process of cancer recurrence, development and evolution. Thus, it has becoming key areas in precision medicine regarding to designing optimal therapeutic approaches targeting the mechanisms and phenotypes of tumor heterogeneity, based on novel detecting techniques and new concepts and theory. This review summarized the current references on tumor heterogeneity, in order to better understand the function and mechanisms, and eventually manage tumor heterogeneity through various methods.

同病异治和异病同治体现了早期人们对肿瘤异质性的认知，随着大规模基因测序等技术手段和对肿瘤生物起源和发展的了解^[1-4]^，有关肿瘤异质性的新研究和新概念不断涌现，包括伴随治疗而产生的肿瘤时间异质性和通过克隆进化而体现的肿瘤空间异质性^[5]^。肿瘤异质性不仅表现在肿瘤内和肿瘤间、原发和继发肿瘤、肿瘤细胞和循环肿瘤细胞存在异质性，也表现在同一肿瘤组织的不同肿瘤细胞间存在异质性。根据肿瘤异质性设计更精准的药物组合，才能发挥最大药物有效性和产生最小药物毒性^[6]^。本文主要围绕近年来肿瘤异质性在发展历程、驱动因素、研究现状、检测手段以及治疗策略方面的最新进展进行综述。

## 1 肿瘤异质性的发展历程

最初发现肿瘤具有异质性可追溯到19世纪，病理学之父Virchow在光镜下发现癌细胞具有不同形态，这也是肿瘤进行病理分型的基础^[7]^。在20世纪50年代，Makino等^[8]^通过研究单细胞水平的细胞遗传学特征和致瘤性，发现自发性肿瘤存在功能和遗传基因的差异，验证了肿瘤存在异质性。70年代-80年代涌现了不同的假说解释肿瘤异质性的形成机制。Nowell等^[9]^认为连续多轮克隆选择是导致肿瘤基因及其他分子变异的根本原因，并绘制了癌症的克隆进化模型。Harris等^[10]^则提出“动态异质性”假说。研究者发现，在小鼠肉瘤中产生的转移性亚克隆的产生比率比耐药性稳定突变的发生率高出10倍-1, 000倍，在某些情况下亚克隆获得转移潜能是可逆的。2005年后随着下一代测序（next-generation sequencing, NGS）的应用，人们对肿瘤异质性的认识提升到了基因层面，在诸如恶性胶质瘤、乳腺癌、结肠癌、肾癌、胰腺癌、卵巢癌等中对肿瘤异质性进行分析，尤其是对于乳腺癌单细胞的测序，证实了肿瘤组织中存在不同类型的亚克隆，不同患者可能存在通用克隆型，不同癌种的克隆型数目不等^[11]^。

## 2 肿瘤异质性产生学说

### 2.1 基因组不稳定性

基因组不稳定性是许多癌症发生发展的源头^[12, 13]^。DNA异常或损伤可引起S期DNA无法修复，中心体异位扩增，纺锤体组装检查点异常，导致复杂的染色体重排，包括基因丢失、扩增和易位，最终引起肿瘤的基因异质性^[14]^。这种不稳定性可能是由于暴露于外源性突变源（如紫外线辐射或烟草烟雾）和内源性过程中的畸变（如DNA复制和/或修复错误或氧化应激）所引起的^[15, 16]^。大规模基因组测序的研究能够发现一些与诱变特征相关的遗传性特征。例如，与吸烟有关的肺癌含有大量C > A颠换，同样，错配修复（mismatch repair, MMR）缺陷的结直肠癌也容易发生C > T转换^[4, 17, 18]^。此外，尽管化疗对基线基因组不稳定性没有影响，但可能扩大肿瘤的突变图谱并造成基因组不稳定性^[19-21]^。

某些理论认为，肿瘤的发生依赖于自发性突变率的^[22, 23]^，癌症往往共同选择内源性内稳态过程，以增加总的突变负荷。例如，在大约一半的人类癌症中由DNA dC→dU编辑酶APOBEC3B上调产生的DNA胞嘧啶脱氨基有助于突变的发生^[24-26]^。TpC位点C > T和C > G突变的APOBEC富集于肿瘤发展的晚期阶段，在细胞毒性化疗后较为普遍^[26-28]^。APOBEC3B高水平表达预示某些癌症患者的预后较差^[29, 30]^。此外，APOBEC突变可导致参与治疗抵抗的基因发生突变。因此，对于这些酶的有效抑制可能会降低遗传不稳定性，从而改善患者预后^[26]^。

ECDNA是一种环状染色体外DNA，这种编码致癌基因的ECDNA存在于许多肿瘤细胞中，可推动肿瘤异质性。对117个来自患者的癌细胞株、8个正常组织以及10个永生细胞株进行检测，发现40%的癌细胞株以及近90%的患者脑肿瘤模型中能够检测到ECDNA，其含量存在差异性，而ECDNA在正常组织中几乎不存在^[31]^。与染色体相比，致癌基因*EGFR*和*c-MYC*在ECDNA上的拷贝数显著增多，ECDNA的不均等分离能够快速增加异质性并使其持续保持在较高水平^[31]^。

### 2.2 克隆进化/选择假说

个体肿瘤的特定区域和不同转移位点中基因组不稳定性的证据表明，基因组不稳定性促进了更具竞争性的亚克隆的出现^[5, 32, 33]^。肺癌治疗前存在耐药克隆，经TKI治疗，敏感克隆减少，耐药克隆逐渐占优势，出现肿瘤异质性^[34]^。然而，过度的基因组不稳定性会对癌细胞的生存和适应性产生不良影响^[35, 36]^。

目前对于肿瘤进化主要遵循克隆进化和/或选择框架模型进行研究。这种肿瘤进化模型中，线性进化描述了由于连续获得赋予生长和/或存活优势的突变而产生的进化，其中顺序克隆包含这些有利的突变，对比祖先克隆更具优势。分支进化显示出现不同增殖的多个亚克隆肿瘤细胞群体存在共同祖先。分枝进化使产生更多异质性肿瘤的可能性大大增加^[37-39]^，如同一*EGFR*突变的非小细胞肺癌（non-small cell lung cancer, NSCLC）肿瘤经TKI治疗，可出现不同的耐药克隆^[34]^。许多实体肿瘤采用分支的进化模式，而某些血液系统恶性肿瘤的系统发育则采用线性模式^[38-43]^。不过，一些研究对克隆亚群必须始终处于竞争中的假设提出质疑，这些研究结果显示，在非细胞自主启动的癌症中，不同亚克隆共同促进肿瘤细胞增殖^[44-46]^。此外，干细胞学说研究发现，肿瘤干细胞在克隆进化过程中存在不同模式，某些肿瘤干细胞在克隆进化的过程中发生突变，增加肿瘤异质性；某些肿瘤干细胞再分化为非肿瘤干细胞的过程中发生突变，增加异质性；还有些肿瘤干细胞发生突变，在随后的进化和分化过程中产生异质性。因此不同的肿瘤干细胞模式对肿瘤耐药及疾病进展可产生不同影响^[47]^。

## 3 肿瘤异质性的研究现状

当今对于肿瘤异质性的研究正在如火如荼地进行。Gerlinger等^[48]^发现，对同一个原发性肾脏肿瘤患者的几个位点及其几个转移瘤进行活组织检测，在鉴定出的突变中只有34%的突变在所有样品中一致，因此对肿瘤的一个区域进行活检并不能很好地描绘出在某个肿瘤发病中起重要作用的癌基因。但另一项研究对11例手术切除局限性肺腺癌的48个肿瘤区域进行了全外显子组测序发现，在7, 269个突变中有76%的突变以及21个已知癌症基因突变中的20个突变基因存在于同一肿瘤的所有区域^[49]^。因此肿瘤异质性在不同癌症类型之间存在差异。

随着国内外研究者对肿瘤异质性研究的不断深入，其研究领域已经从细胞及动物学研究逐步过渡到临床试验研究。TRACERx对招募842例早期肺癌（Ⅰa期-Ⅲa期）患者手术切除的NSCLC肿瘤样本进行高深度、多区域全外显子测序，发现100例患者的327块肿瘤组织的细胞之间存在广泛异质性，包括中位数为30%的体细胞突变以及中位数为48%的拷贝数改变。具有高比例亚克隆拷贝数变化（≥48%，队列中位数）的肿瘤患者复发或死亡的风险高于比例低的患者。一半左右的亚克隆驱动基因改变与基因组稳定性相关^[32]^。另一项针对TRACERx研究显示，在所有46例ctDNA阳性患者中，平均有27%的亚克隆SNVs被检出，揭示了患者主克隆和亚克隆突变具有异质性。此外，肿瘤的亚克隆演化过程揭示了其肿瘤时间异质性。对组织标本原发肿瘤5个区域病灶（手术），复发（第467天），死亡（第857天）以及血液标本术后连续采血（术后60天、151天、242天、340天、431天、466天、627天、767天）进行分析发现，克隆性变异等位基因频率（variant allele frequency, VAF）与肿瘤大小相关^[50]^。

## 4 肿瘤异质性的检测技术

人们对于肿瘤异质性认识的不断深入，其根本原因是检测技术的迅速发展与不断进步。目前，人们可通过数字PCR（digital PCR）和NGS等技术手段实现对循环肿瘤细胞（circulating tumor cells, CTCs）、血液中的游离DNA（cell-free DNA, cfDNA）、循环肿瘤DNA（circulating tumor DNA, ctDNA）和肿瘤组织DNA的检测。dPCR是一种核酸分子绝对定量技术，能够对起始样本的进行绝对定量。dPCR测序深度较高，可以定量分析低至0.001%-0.000, 1%的突变频率，但基因检测种类有限。相比之下，NGS可实现大规模平行测序，能够同时对数百万个DNA片段进行测序。通过对同一患者的一个或多个病灶的多区域样本测序让人们从基因水平认识到了肿瘤存在空间异质性^[20, 40, 48-50]^。因此，对组织进行多区域测序可以在空间上有效地对肿瘤的进化进行动态观察。肿瘤空间和时间异质性变化能够描述克隆进化过程，对肿瘤异质性遗传改变进行实时追踪可以用来分析肿瘤的克隆演进^[50]^。

对早期肺腺癌患者的CTCs水平进行检测发现，早期肺腺癌中可检测到上皮型、混合型、间质型三种类型CTCs，体积较大的肿瘤倾向于产生更多间质型CTCs。此外，对CTCs进行连续检测能够有效预测疾病动态^[51]^。分离自血浆和其他生物液体的ctDNA可用来描述肿瘤基因表征。而cfDNA的半衰期约为2 h^[52]^，可以实时监测等位基因频率的变化。液态活检可以检测到克隆和亚克隆的变化：对进行多区域活组织检查取样的早期NSCLC患者有效地进行了"系统性ctDNA追踪"，并且通过液态活检对代表性的单核苷酸变体的躯干和分支突变进行纵向监测^[50, 53]^。这表明手术时多区域活检分析（空间异质性）中获得的信息在一定程度上可以转化为ctDNA分析。介导特定靶向药物耐药的相对频率（等位基因分数）的增加可用于检测难治性肿瘤亚群（分支）的进化。ctDNA数量的差异与肿瘤的组织学类型、部位、分期有关，另外在原发性NSCLC中ctDNA数量也与坏死和代谢活性有关。此外，一些原发性肿瘤难以进行活检，临床医生通常依靠细针穿刺活检（fine-needle aspiration biopsy, FNA）来取得肿瘤组织进行组织病理学检查和诊断^[54]^。但FNA并不总是产生足够的材料进行批量深度测序，当生成测序文库时，其复杂性也很低，这反过来影响了亚克隆变体发现^[55]^。因此，单细胞测序将有助于分析这些肿瘤，从而高分辨率解读肿瘤异质性。

## 5 肿瘤异质性的治疗策略

### 5.1 调节基因组不稳定性

基因组不稳定是导致肿瘤异质性的原因之一。目前人们仅部分了解控制基因组稳定性的因素。例如DNA同源重组缺陷和错配修复缺陷是常见的与导致基因组不稳定的因素。BRCA是DNA损伤同源重组修复中的重要成分，而PARP在单链DNA损伤修复中起重要作用的分子，同时抑制针对两条损伤修复途径可以发挥协同致死作用。PARP抑制剂治疗存在BRCA1及BRCA2缺陷的肿瘤就是最常见的调节基因组不稳定性的治疗策略^[56]^。此外，研究^[57]^发现，高突变负荷的肿瘤对免疫检查点抑制剂更敏感。携带错配缺陷的肿瘤，增加基因组的不稳定，增加肿瘤的突变负荷，已经成为筛选病人免疫靶向治疗的标志物。FDA已经批准Keytruda治疗存在错配修复缺陷的全肿瘤。最近有报道通过烷化剂例如替莫唑胺治疗可以诱导患者出现微卫星不稳定性，增加免疫检查点治疗的疗效，延缓肿瘤进展，降低异质性的发生^[58]^。另外，在预防性治疗和辅助治疗中通过影响基因组不稳定性，将最大程度改善患者的预后，延缓肿瘤进展。

### 5.2 靶向克隆性驱动基因突变

在肿瘤进化的过程中，选择压力促进肿瘤细胞在主克隆之外出现一些亚克隆改变，从而产生异质性并驱动肿瘤进展。克隆性驱动基因突变存在于绝大多数肿瘤细胞的克隆性突变中，靶向克隆性驱动基因突变是持续的控制肿瘤的策略。*EGFR*和*ALK*是NSCLC常见的驱动突变，是驱动NSCLC发生发展的主干突变。针对这两个靶点的治疗已成为存在驱动突变NSCLC的标准治疗选择，可使NSCLC患者预后显著延长。例如一代TKI药物针对EGFR常见敏感突变有效，阿法替尼对常见*EGFR*敏感突变和一些少见突变如G719X、E709X、Ins19是敏感的，而第三代TKI对敏感突变，T790M突变有效。*ALK*融合突变存在不同重排方式，基础研究发现，每种不同的ALK抑制剂仅对部分ALK重排敏感，通过NGS测序确定重排类型进行选择。

### 5.3 组合疗法

靶向克隆性突变的单一药物难以根治肿瘤，从不同层面抑制肿瘤细胞的两种或两种以上药物的组合疗法可能是更有效的治疗策略。例如*EGFR*突变NSCLC在TKI在治疗过程中，随着药物的选择压力，不断有*EGFR*新突变的发生，也不断有原有*EGFR*突变的消失。C797S是第三代TKI单药治疗常见耐药机制，但C797S与T790M突变呈反式给予患者厄洛替尼+奥希替尼治疗是克服耐药的策略。不过目前毒性是联合治疗应用的主要限制。

### 5.4 预防性组合疗法

在治疗开始时，耐药细胞克隆通常是存在的，早期的联合治疗根除这种克隆的可能性较大。然而，肿瘤细胞与宿主之间狭窄的治疗窗口限制了可同时联合的药物的数量。靶向药物的预防性联合旨在同时靶向大多数肿瘤（药物在躯干突变上的活化）和预期的继发耐药机制，因此与复发时给药相比，预防性组合疗法对于肿瘤存活率的影响具有显著优势^[59]^。联合抑制EGFR/MEK可以预防*EGFR*突变肺癌模型耐药的出现^[60]^。Crystal等^[61]^建立了一个获得性耐药患者衍生模型的平台，用于鉴定有效的靶向药物组合。结果显示，与耐药组合相比，预防性组合疗法显著增加的应答。一项晚期NSCLC患者二期临床试验^[62]^显示，对于治疗前就存在*EGFR*耐药突变（T790M）的患者，厄洛替尼联合贝伐单抗的中位PFS为16.0个月，1年无进展生存率为68%。

预防性组合疗法的局限性主要是患者耐药机制的变异性。对MAPK抑制剂失去敏感性的转移性黑素瘤中，不仅BRAF、NRAS、KRAS、MEK1以及MAP2K1的变异在复发时富集，并且激活了不同的逃逸信号通路，如PIK3CA、AKT1和AKT3功能获得；PIK3R2、DUSP4、CDKN2A、PTEN的功能缺失以及非基因组改变如MET过表达和β-catenin和YAP1失调^[63]^。

### 5.5 免疫疗法来靶向主干基因突变

影响靶向药物多重克隆改变受药物和毒性问题的限制。克服这些局限性的策略包括通过手术、系统治疗、个体化疫苗或过继性细胞疗法靶向克隆新抗原或主要分支抗原^[64]^。通过这些方法靶向多种新抗原可显着降低耐药性。Wu等^[65]^为每例黑色素瘤患者制作了13种-20种含有新抗原的免疫多肽疫苗。结果显示，在接种疫苗的6例患者中，4例在接种疫苗后25个月没有复发。另一组研究设计了针对于黑色素瘤患者进行个体化RNA疫苗治疗的全套方案，包括全面识别个体突变，新表位的计算预测，以及为每个病人设计和制造特异性疫苗（不超过10种不同的编码新抗原的RNA片段）。接种疫苗后，患者转移累积速率显着降低，在13例黑色素瘤患者中，8例患者在接受疫苗后1年内没有出现复发迹象，5例转移性疾病患者中有2例接种疫苗后肿瘤明显缩小，另外1例患者在接受PD-1抑制剂联合疫苗治疗后得到完全缓解^[65]^。

### 5.6 适应性疗法

在特定的治疗压力选择下，某些耐药性克隆存在克隆选择优势，成为推动疾病进展的主要因素，当特定的治疗选择压力消失时，药物敏感型克隆与耐药克隆之间可保持增殖的平衡。因此，可以通过动态监测肿瘤在治疗过程的适应性克隆演变帮助决定暂停治疗和重新开始治疗的时机。

### 5.7 免疫表型异质性的治疗策略

肿瘤的免疫表型主要包括免疫炎症型，免疫豁免型及免疫沙漠型。对于不同的免疫表型，常采用不同的治疗策略。

免疫炎症型，肿瘤组织内部和周围有大量的免疫细胞侵润。对于CD8^+^淋巴细胞浸润并有功能的肿瘤患者，常采用PD-1/PD-L1抑制剂单药治疗；而对于CD8^+^淋巴细胞浸润并无功能的肿瘤患者，可以尝试PD-1/PD-L1抑制剂联合MEK抑制剂或IDO1抑制剂的治疗策略。

免疫豁免型，肿瘤组织周围存在棉衣细胞浸润，不能穿透肿瘤的实质，而是保留在围绕肿瘤细胞巢的基质中^[66-69]^。用抗PD-L1/PD-1药物治疗后，基质相关的T细胞活化和增殖，但不会浸润，临床应答不常见。因此，可能预先存在的抗肿瘤反应，但是由于通过基质的肿瘤穿透阻滞或免疫细胞在间质中的滞留而使其变得无效。目前针对与免疫豁免型肿瘤的治疗主要采用PD-1/PD-L1抑制剂联合抗血管治疗。通过肿瘤基质的T细胞迁移是这种免疫豁免型肿瘤免疫循环中的限速步骤。

免疫沙漠型，肿瘤组织内部和周围都缺少免疫性浸润^[70, 71]^。抗PD-L1/PD-1治疗对这种类型的肿瘤没有明显效果^[66]^。因此对于免疫沙漠型的肿瘤患者，常采用抗PD-L1/PD-1与其他治疗相结合的策略，包括化疗、放疗、其他靶向药物、疫苗、其他免疫通路抑制剂（CTLA-4抑制剂、TIM3阻断剂、Lag3抑制剂）。这种表型可能反映了没有预先存在的抗肿瘤免疫力，表明肿瘤特异性T细胞的产生是限速步骤。

## 6 总结与展望

异质性是肿瘤普遍存在的现象，由于其导致的肿瘤耐药使肿瘤治疗陷入困境。随着多区域活检、液体活检、单细胞测序等检测技术的不断进步，肿瘤异质性逐渐被揭示，人们对于肿瘤异质性的认识也在不断深入。通过对肿瘤异质性发生机制的研究，克服治疗中的治疗有效-耐药-再治疗-再耐药的循环是肿瘤治疗中的重中之重。因此明确肿瘤间，肿瘤内部时空异质性，根据每个肿瘤每个时期给予分层靶向治疗才能真正实现精准医疗。
